# The association between leptin, adiponectin levels and the ovarian reserve in women of reproductive age

**DOI:** 10.3389/fendo.2024.1369248

**Published:** 2024-05-17

**Authors:** Konstantinos Nikolettos, Nikolaos Vlahos, Olga Pagonopoulou, Nikos Nikolettos, Konstantinos Zikopoulos, Panagiotis Tsikouras, Emmanouil Kontomanolis, Christos Damaskos, Nikolaos Garmpis, Iason Psilopatis, Byron Asimakopoulos

**Affiliations:** ^1^ Obstetric and Gynecologic Clinic, Medical School, Democritus University of Thrace, Alexandroupolis, Greece; ^2^ Second Department of Obstetrics and Gynecology, National and Kapodistrian University of Athens, School of Medicine, Aretaieion Hospital, Athens, Greece; ^3^ Laboratory of Physiology, Faculty of Medicine, Democritus University of Thrace, Alexandroupolis, Greece; ^4^ Department of Obstetrics and Gynecology, University Hospital of Ioannina, Ioannina, Greece; ^5^ Renal Transplatation Unit, Laiko General Hospital, Athens, Greece; ^6^ NSChristeas Laboratory of Experimental Surgery and Surgical Research, Medical School, National and Kapodistrian University of Athens, Athens, Greece; ^7^ Department of Surgery, Sotiria General Hospital, Athens, Greece; ^8^ Department of Gynecology and Obstetrics, Universitätsklinikum Erlangen-Frauenklinik, Erlangen, Germany

**Keywords:** leptin, adiponectin, ovarian reserves, polycystic, reproductive age, ovarian, syndrome

## Abstract

**Background:**

Reproduction ability requires a certain amount of body fat that is necessary for ovulation, menstruation and pregnancy. Fat tissue represents an endocrine organ with high metabolic activity as it produces adipokines such as leptin and adiponectin. Our aim is to examine potential associations between women of reproductive age’s ovarian reserves and their levels of leptin and adiponectin.

**Method:**

74 women between 19 and 40 years of age consented to take part. Based on the patterns of their ovarian reserves, the women were divided into three main groups: women with adequate ovarian reserves (AOR - Group A, n=30), women with polycystic ovary syndrome (PCOS - Group B, n=31) and women with depleted ovarian reserves (DOR - Group C, n=13). Among these groups, several biochemical and demographic parameters were statistically compared.

**Results:**

Compared to the other two groups, women with DOR had statistically higher age and follicle stimulation hormone (FSH) levels. For estradiol (E2) and thyroid-stimulating hormone (TSH), no statistically significant difference was seen between the groups. In addition, women with PCOS had higher body mass index (BMI), luteinizing hormone (LH), total testosterone (TT), 17 hydroxyprogesterone (17-OHP), dehydroepiandrosterone (DHEA), anti-Mullerian hormone (AMH), and antral follicle count (AFC) than the other two groups. In line with expectations, women with DOR also had lower levels of AMH and AFC than the other two groups. Women with PCOS had higher leptin levels than the other two groups, but there was no statistically significant difference. Women with PCOS had lower levels of adiponectin than the other groups, however the difference was not statistically significant.

**Conclusion:**

The way we classified women in our study according to their ovarian reserves is completely consistent with what has been published internationally. The ovarian reserve in women of reproductive age is not strongly correlated with leptin and adiponectin levels. For safe conclusions, more research including a greater number of samples is required.

## 1 Introduction

In 1974, Frisch and his team reported the ability to reproduce requires a certain amount of body fat that is necessary for ovulation, menstruation and pregnancy (1). Adipose tissue represents a dynamic organ with metabolic role and it contains adipocytes. It produces adipokines such as leptin and adiponectin (2).

Leptin plays crucial role in regulating female reproductive health through distinct molecular pathways. Leptin, primarily secreted by adipose tissue, acts on the hypothalamus to stimulate the release of gonadotropin-releasing hormone (GnRH), thereby influencing the menstrual cycle and fertility (3–5). Leptin concentrations are directly related to body fat content (6). Furthermore, leptin levels are very sensitive to caloric deprivation (7). Leptin is a 167 amino acid polypeptide that is mostly produced by fat cells but is also found in the placenta, mammary glands, and stomach (8). Moreover, leptin levels are at their lowest at 09:00 and reach their peak at 01:00 (9). Serum leptin levels are strongly lowered during fasting, even before weight decrease, despite the fact that they are positively correlated with adipose tissue (10). Despite the fact that leptin inhibits appetite, obese individuals have higher serum leptin levels and may be leptin resistant, which is comparable to the insulin resistance that is frequently observed in obesity (11). Serum leptin levels are also influenced by steroids, fat distribution, and gender (12). Additionally, subcutaneous and visceral adipose tissue express leptin differently (13).

The most widely released adipokine that is only expressed in adipose tissue is called adiponectin (2, 14). αdiponectin modulates reproductive function by affecting ovarian steroidogenesis and folliculogenesis, contributing to the regulation of ovulation and fertility (5). Three distinct forms of adiponectin have been identified: low molecular weight adiponectin, hexamer adiponectin, and high molecular weight adiponectin (15). Adipocyte mass is inversely correlated with circulating adiponectin levels, which rise with weight loss and fall with obesity (16). Men and women have different levels of adiponectin; women typically have higher levels of this hormone (17). Rats, chickens, pigs, cows, and other animals have also been shown to express adiponectin and its receptors in their ovarian granulosa cells (18–20).

These aforementioned adipokines serve as key links between energy metabolism and reproductive processes, highlighting the intricate interplay between adipose tissue and reproductive physiology. Suboptimal reproductive health, such as irregular menstrual cycles or polycystic ovarian syndrome (PCOS), can disrupt the balance of hormones involved in metabolism, including leptin and adiponectin. In conditions like PCOS, alterations in insulin sensitivity and ovarian function can lead to elevated leptin levels and decreased adiponectin levels, contributing to metabolic disturbances and further exacerbating reproductive dysfunction (3–5).

In the present study, we investigated whether the circulating levels of leptin and adiponectin correlate with the ovarian reserves.

## 2 Methods

### 2.1 Study design

Between February 2020 and November 2022, a prospective cross-sectional study was carried out in the Laboratory of Physiology, Faculty of Medicine, Democritus University of Thrace, in cooperation with the IVF clinic “Embryokosmogenesis,” in Alexandroupolis, Greece. The Democritus University of Thrace’s Research Ethics Committee (34792/223/13-2-2020) gave its approval to the study. Following a thorough explanation of the study’s objectives, all female participants signed a written consent form. Pregnancy, menopause, smoking, hypo- or hyperthyroidism, endometriosis, hysterectomy, oophorectomy, congenital adrenal hyperplasia, Cushing syndrome, and hypogonadotropic hypogonadism were the exclusion criteria.

**Figure 1:**
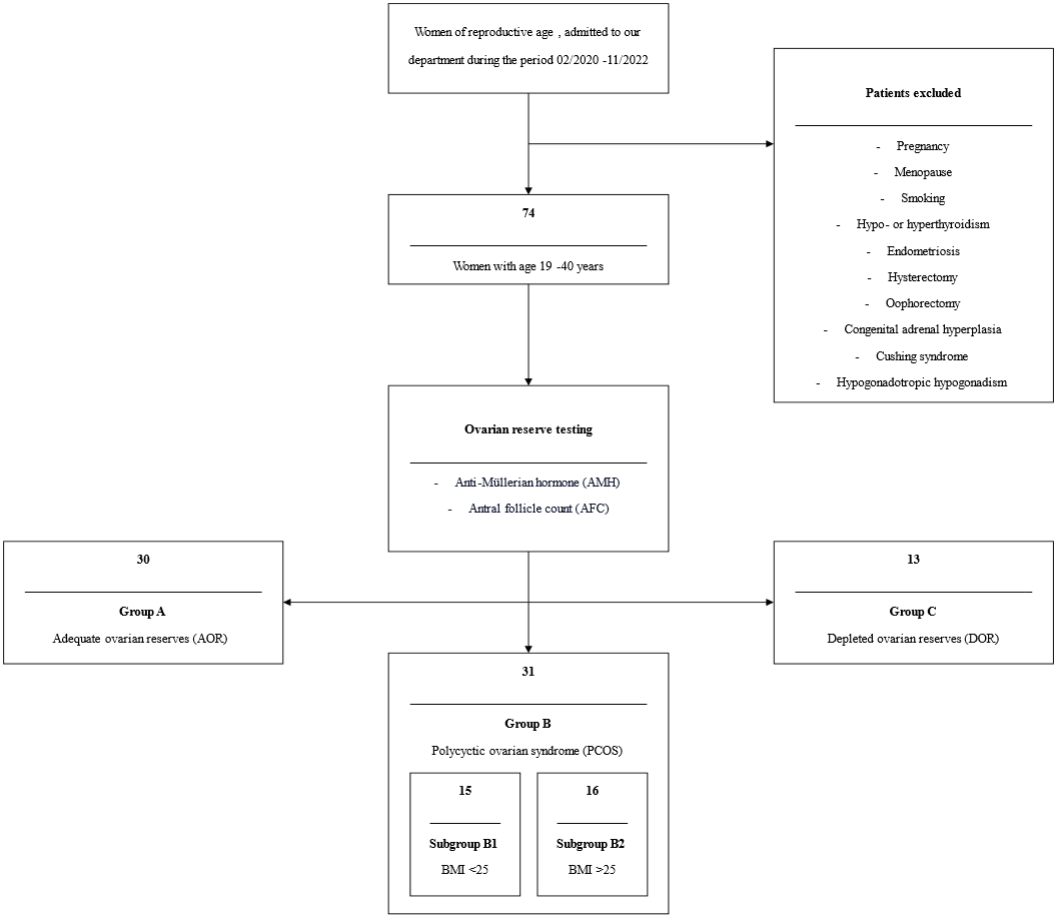
Patient’s selection criteria and the flow chart of the current study. AMH, Anti-Müllerian hormone; AFC, Antral follicle count; AOR, Adequate ovarian reserves; PCOS, Polycystic ovarian syndrome; BMI, Body mass index; DOR, Depleted ovarian reserves.

Seventy-four women, ranging in age from 19 to 40 years, were enlisted. On these women ovarian reserve test was calculated by measuring anti-Mullerian hormone (AMH) and antral follicle count (AFC). Based on these results, these women were divided into three main groups: those with adequate ovarian reserves (AOR) (Group A - AOR, n=30), those with PCOS (Group B - PCOS, n=31), and those with depleted ovarian reserves (DOR) (Group C - DOR, n=13). PCOS was diagnosed using the Rotterdam criteria (21). In accordance with the Committee Opinion no 618 of the American College of Obstetricians and Gynecologists, diminished ovarian reserves were defined as <7 follicles in both ovaries (22). **Figure 1** indicates the patient selection criteria and the flow chart of the current study.

Women were first examined physically, with measurements taken for height, weight, and body mass index (BMI). On days 2–5 of the menstrual cycle, the same medical professional calculated the AFC using a Voluson 730P (General Electric Co.) ultrasound machine fitted with a vaginal transducer. Between day two and day five of the menstrual cycle, blood samples were drawn once for the purpose of measuring the following: follicle stimulation hormone (FSH), luteinizing hormone (LH), estradiol (E2), total testosterone (TT), 17 hydroxyprogesterone (17-OHP), dehydroepiandrosterone (DHEA), thyroid-stimulating hormone (TSH), AMH, leptin and adiponectin. After clotting at room temperature for ten minutes, blood samples were centrifuged at 1500 g for the purpose of extracting serum. Every day, serum samples were examined for FSH, LH, 17-OHP and E2. Serum samples were frozen at -80°C for the leptin, adiponectin, and AMH measurements within an hour of blood being drawn. ELISA was used to measure the serum levels of leptin and adiponectin using the following kits as per the manufacturer’s instructions: leptin (DLP00, R&D, USA) and adiponectin (DRP300, R&D, USA).

Finally, the biochemical and demographic traits of the three groups under investigation are presented in **Table 1**.

**Table 1:** Demographic and biochemical parameters of the three groups under investigation.

Variables	Group A (AOR) n=30	Group B (PCOS) n=31	Group C (DOR) n=13	*P* value
BMI (kg/m^2^)	22.71 ± 4.62	27.35 ± 6.57	22.6 ± 2.28	0.0031*
Age	30.03 ± 6.36	27.26 ± 5.11	35.69 ± 4.62	<0.001*
AFC	15.43 ± 5.07	31.7 ± 7.24	5.69 ± 1.6	<0.001*
FSH (IU/L)	5.33 ± 1.52	4.68 ± 1.17	8.47 ± 1.4	<0.001*
LH (IU/L)	4.33 ± 1.45	8.19 ± 5.2	4.12 ± 2.59	<0.001*
E2 (pg/mL)	56.13 ± 26.55	50.57 ± 26.32	62.69 ± 23.9	0.08
TT (ng/dL)	0.39 ± 1.35	0.74 ± 0.38	0.4 ± 0.22	<0.001*
17-OHP (ng/dL)	0.81 ± 0.39	1.33 ± 0.82	0.78 ± 0.38	0.0134*
DHEA (mcg/dL)	4.57 ± 1.6	5.9 ± 2.75	3.96 ± 1.84	0.0174*
TSH	1.7 ± 0.74	2.19 ± 1.54	1.81 ± 1.13	0.479
AMH (ng/dL)	2.24 ± 0.65	7.59 ± 2.66	0.86 ± 0.42	<0.001*
Leptin (μg/ml)	6.3 ± 9.5	9.17 ± 9.88	8.58 ± 10.86	0.177
Adiponectin (μg/ml)	3.82 ± 3.07	3.23 ± 2.55	3.38 ± 2.52	0.77

Values are expressed as the mean ± standard deviation.

*p values indicate statistically significant (p<0.05).

AOR, Adequate ovarian reserves; PCOS, Polycystic ovarian syndrome; DOR, Depleted ovarian reserves; BMI, Body mass index; AFC, Antral follicle count; FSH, Follicle-stimulating hormone; LH, Luteinizing hormone; E2, Estradiol; TT, Total testosterone; 17OHP, 17-hydroxy progesterone; DHEA, Dehydroepiandrosterone; TSH, thyroid-stimulating hormone; AMH, Anti-Mullerian hormone.

### 2.2 Statistical analysis

The statistical software Statistica 8.0 (StatSoft Inc., Tulsa, OK, USA) was used to conduct the statistical analysis. Utilizing the Kolmogorov Smirnov and Shapiro-Wilks tests, the normality of the data was examined. Since some of the data did not fit into a normal distribution, group comparisons were made using non-parametric tests. The levels of adiponectin and leptin were tested for any significant correlation with the other parameters using Spearman rank R. A threshold of 0.05 was established for statistical significance.

## 3 Results

**Figure 2:**
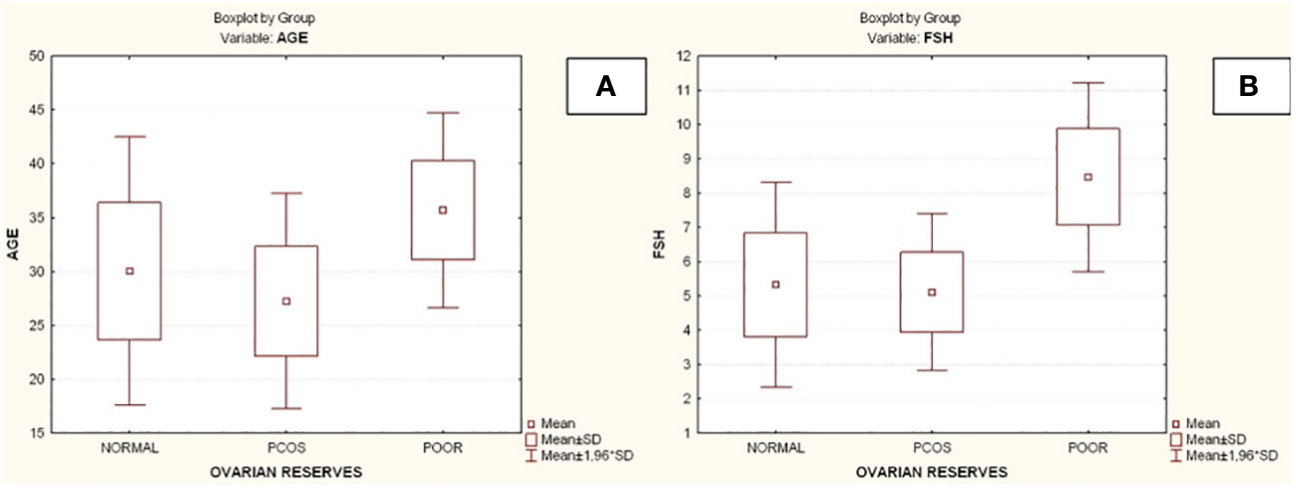
Comparison of parameters and ovarian reserves in all groups. **(A)** Age; **(B)** Follicle-stimulating hormone (FSH). PCOS, Polycystic ovarian syndrome; SD, Standard deviation; FSH, Follicle-stimulating hormone.

**Figure 3:**
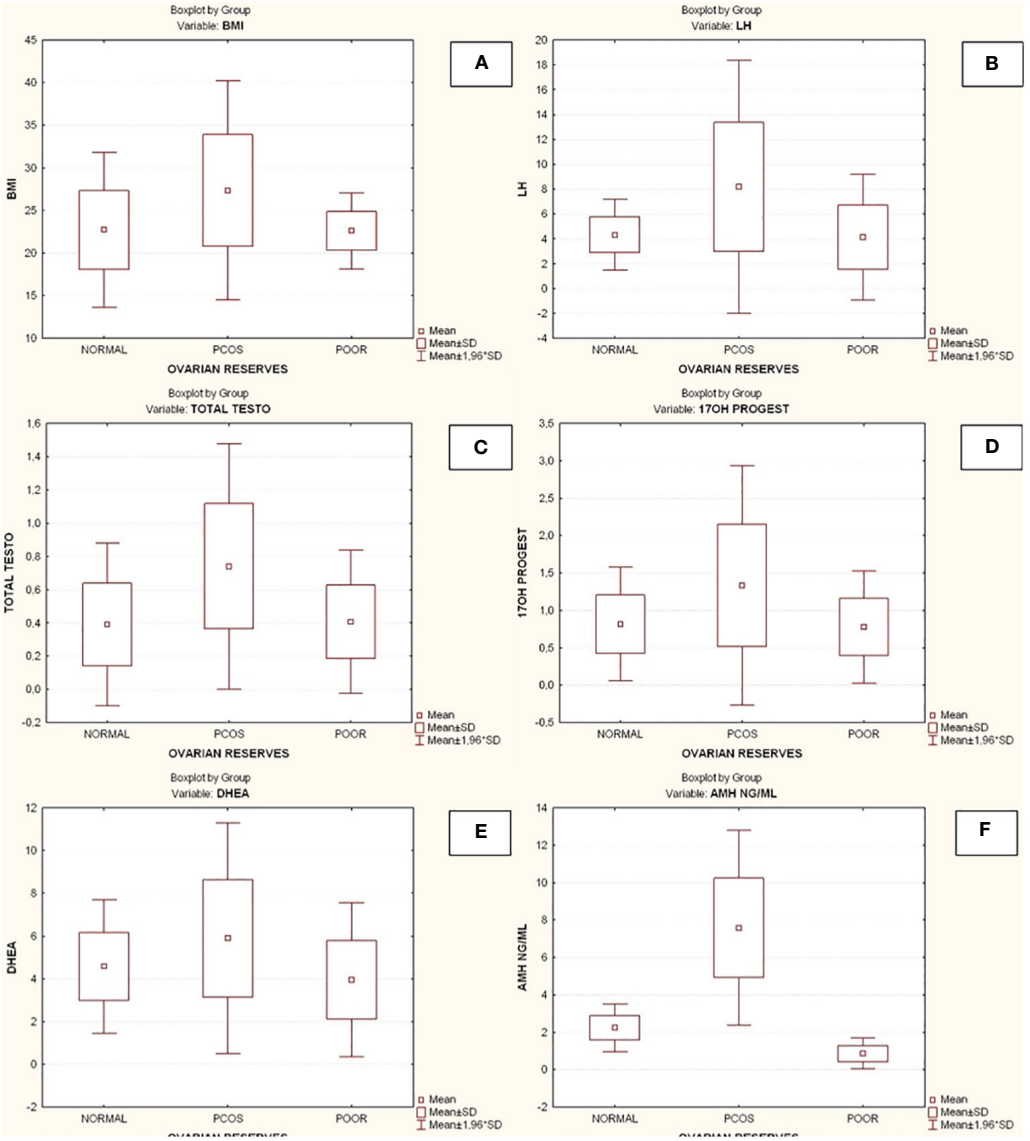
Comparison of parameters and ovarian reserves in all groups. **(A)** Body mass index (BMI); **(B)** Luteinizing hormone (LH); **(C)** Total testosterone (TT); **(D)** 17-hydroxy progesterone (17OHP); **(E)** Dehydroepiandrosterone (DHEA); **(F)** Anti-Mullerian hormone (AMH). BMI, Body mass index; PCOS, Polycystic ovarian syndrome; SD, Standard deviation; LH, Luteinizing hormone; TT, Total testosterone; 17OHP, 17-hydroxy progesterone; DHEA, Dehydroepiandrosterone; AMH, Anti-Mullerian hormone.

**Figure 4:**
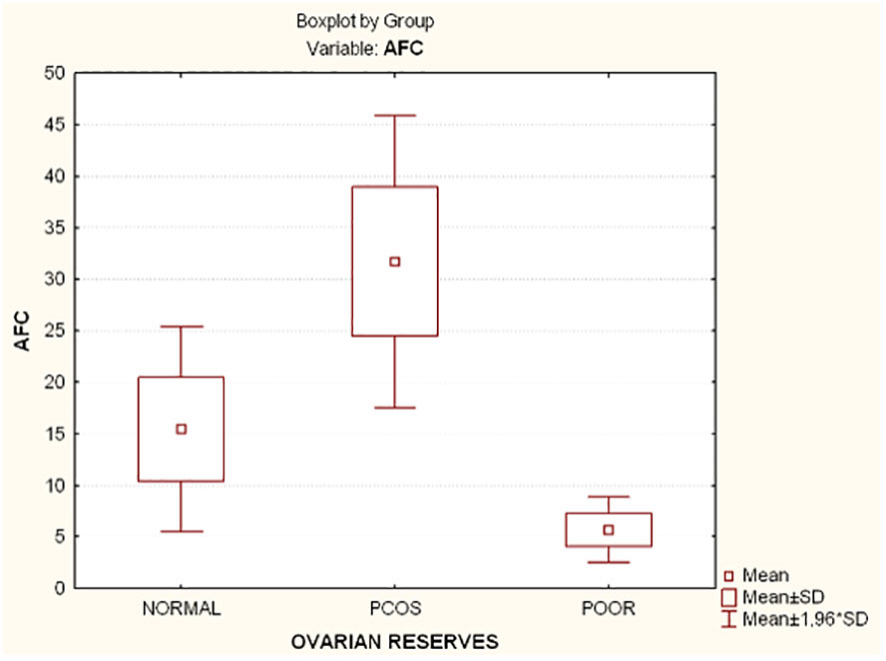
Comparison of antral follicle count (AFC) and ovarian reserves in all groups. AFC, Antral follicle count; PCOS, Polycystic ovarian syndrome; SD, Standard deviation.

**Figure 5:**
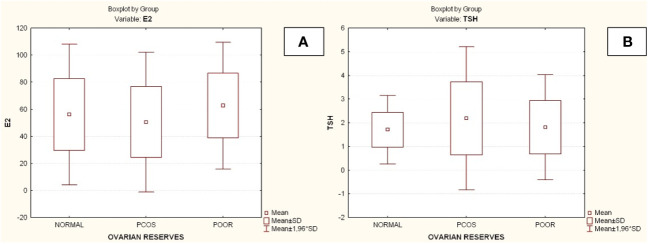
Comparison of parameters and ovarian reserves in all groups. **(A)** Estradiol (E2); **(B)** Thyroid-stimulating hormone (TSH). E2, Estradiol; PCOS, Polycystic ovarian syndrome; SD, Standard deviation; TSH, thyroid-stimulating hormone (TSH).

**Figure 6:**
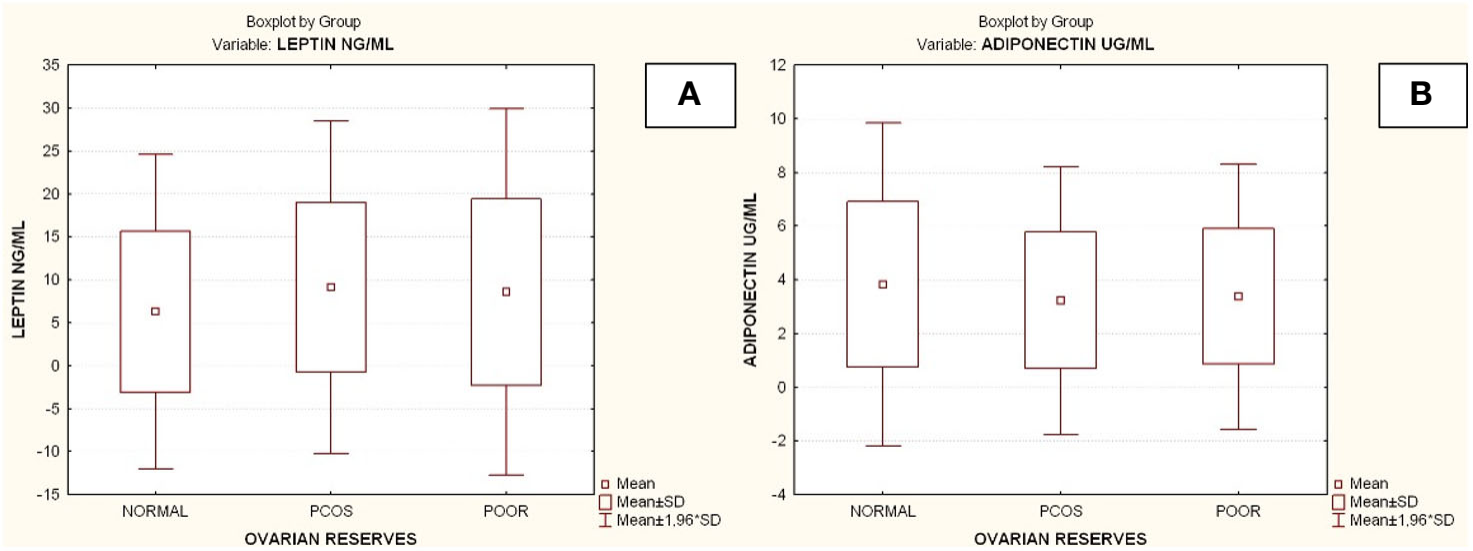
Comparison of parameters and ovarian reserves in all groups. **(A)** Leptin; **(B)** Adiponectin. PCOS. Polycystic ovarian syndrome; SD. Standard deviation. Diagrams Diagram 1. Patient’s selection criteria and the flow chart of the current study. AMH. Anti-Müllerian hormone; AFC. Antral follicle count; AOR. Adequate ovarian reserves; PCOS. Polycystic ovarian syndrome; BMI. Body mass index; DOR. Depleted ovarian reserves.

Compared to the other two groups, the group with lower ovarian reserves (Group C) had statistically significantly higher age and FSH values (Kruskal-Wallis, *p*=0.0031 and *p*<0.001 respectively) (**Figures 2A, B**). In comparison to the other two groups, women with high ovarian reserves (Group B) had higher BMI, LH, TT, 17-OHP, DHEA and AMH (Kruskal-Wallis, *p*=0.0031, *p*<0.001, *p*<0.001, *p*=0.0134, *p*<0.001, *p*<0.001, respectively) (**Figures 3A–F**). In this group, AFC was also higher (Kruskal-Wallis, *p*<0.001) (**Figure 4**). As anticipated, women with low ovarian reserve had lower AMH and AFC compared to the other groups (*p*<0.001, *p*<0.001, respectively) (**Figures 3F**, **4**). The Kruskal-Wallis analysis revealed no statistically significant difference inE2 or TSH between the groups (*p*=0.08 and *p*=0.479, respectively) (**Figures 5A, B**). Women with PCOS had higher leptin levels, but the difference was not statistically significant (Kruskal-Wallis, *p*=0.177) (**Figure 6A**). Additionally, although there was no statistically significant difference, women with PCOS had lower adiponectin levels (Kruskal-Wallis, *p*=0.77) (**Figure 6B**). The aforementioned results are described in **Table 1**.

The correlation analyses of leptin and other parameters were performed in women of all groups. In group A and in group B there was not a correlation between leptin and the other parameters. In group C leptin levels were negative correlated with E2 (r=-0,57, *p*<0.05) and positive correlated with AMH (r =0.65, *p*<0.05) and adiponectin (r=0,59, *p*<0.05) (**Table 2**).

**Table 2:** Spearman’s correlation of leptin and adiponectin with the other parameters in all groups.

Variables	Spearman’s rho					
	Group A (AOR)		Group B (PCOS)		Group C (DOR)	
	*Leptin*	*Adiponectin*	*Leptin*	*Adiponectin*	*Leptin*	*Adiponectin*
BMI (kg/m^2^)	0.12	0.13	-0.11	-0.28	-0.26	0.09
Age	0.15	0.05	-0.04	-0.07	-0.35	0.06
AFC	-0.05	0.05	0.15	0.006	0.22	0.03
FSH (IU/L)	0.14	0.2	0.14	-0.02	-0.01	0.08
LH (IU/L)	0.13	-0.005	0.33	-0.37*	0.55	0.47
E2 (pg/mL)	-0.04	-0.22	0.08	-0.18	-0.57*	-0.46
TT (ng/dL)	-0.17	0.09	-0.06	-0.08	0.43	0.28
17-OHP (ng/dL)	-0.31	-0.05	0.07	-0.4*	0.49	0.1
DHEA (mcg/dL)	0.06	0.14	-0.22	-0.06	0.12	0.62*
TSH	-0.2	-0.13	-0.02	-0.12	-0.13	-0.39
AMH (ng/dL)	0.04	0.01	-0.06	-0.07	0.65*	0.13
Leptin (ng/ml)	–	-0.22	–	0.04	–	0.59*
Adiponectin (μg/ml)	-0.22	–	0.04	–	0.59*	–

*p values indicate statistically significant (p<0.05).

AOR, Adequate ovarian reserves; PCOS, Polycystic ovarian syndrome; DOR, Depleted ovarian reserves; BMI, Body mass index; AFC, Antral follicle count; FSH, Follicle-stimulating hormone; LH, Luteinizing hormone; E2, Estradiol; TT, Total testosterone; 17OHP, 17-hydroxy progesterone; DHEA, Dehydroepiandrosterone; TSH, thyroid-stimulating hormone; AMH, Anti-Mullerian hormone.

Also, the correlation analyses of adiponectin and other parameters were performed in women of all groups. In group A there was not a correlation between adiponectin and other parameters. In group B adiponectin levels were negative correlated with LH (r=-0.37, *p*<0.05) and 17-OHP (r =-0.4, *p*<0.05). Finally, in group C adiponectin levels were positive correlated with DHEA (r=0.62, *p*<0.05) and leptin (r=0.59, *p*<0.05) (**Table 2**).

Moreover, in group B, which included women with increased BMI compared to the other two groups, further statistical analysis was performed. Thus, group B was divided into two subgroups based on BMI: B1 (PCOS with BMI<25, n=15) and B2 (PCOS with BMI>25, n=16). In the comparison of subgroup B1 with B2, no statistically significant difference was found for the values of leptin (Mann – Whitney U Test, *p*=0.95) and adiponectin (Mann – Whitney U Test, *p*=0.54).

Furthermore, leptin was correlated with the parameters in subgroup B1, where there was a statistically significant positive correlation between leptin, FSH (r=0.52, *p*<0.05) and LH (r=0.58, *p*<0.05). In subgroup B2, no statistically significant correlation was found between leptin and the parameters (**Table 3**). In subgroup B1 there was a statistically significant negative correlation between adiponectin, LH (r=-0.53, *p*<0.05) and 17-OHP (r=-0.53, *p*<0.05). In subgroup B2, no statistically significant correlation was found between adiponectin and the parameters (**Table 4**).

**Table 3:** Spearman’s correlation of leptin with the other parameters in group B.

Variables	Spearman’s rho	
	Group B (PCOS)	
	Subgroup B1 BMI <25	Subgroup B2 BMI >25
	*Leptin*	
BMI (kg/m^2^)	-0.09	-0.32
Age	0.04	-0.1
AFC	0.38	-0.04
FSH (IU/L)	0.52*	-0.18
LH (IU/L)	0.58*	0.07
E2 (pg/mL)	0.45	-0.19
TT (ng/dL)	0.05	-0.16
17-OHP (ng/dL)	0.29	-0.1
DHEA (mcg/dL)	-0.4	-0.2
TSH	0.09	-0.1
AMH (ng/dL)	0.17	-0.3
Adiponectin (μg/ml)	-0.26	0.27

*p values indicate statistically significant (p<0.05).

PCOS, Polycystic ovarian syndrome; BMI, Body mass index; AFC, Antral follicle count; FSH, Follicle-stimulating hormone; LH, Luteinizing hormone; E2, Estradiol; TT, Total testosterone; 17OHP, 17-hydroxy progesterone; DHEA, Dehydroepiandrosterone; TSH, thyroid-stimulating hormone; AMH, Anti-Mullerian hormone.

**Table 4:** Spearman’s correlation of adiponectin with the other parameters in group B.

Variables	Spearman’s rho	
	Group B (PCOS)	
	Subgroup B1 BMI <25	Subgroup B2 BMI >25
	*Adiponectin*	
BMI (kg/m^2^)	-0.33	-0.4
Age	0.02	-0.07
AFC	-0.17	0.11
FSH (IU/L)	-0.05	0.003
LH (IU/L)	-0.53*	-0.19
E2 (pg/mL)	-0.26	-0.002
TT (ng/dL)	0.11	-0.2
17-OHP (ng/dL)	-0.53*	-0.32
DHEA (mcg/dL)	-0.18	-0.05
TSH	0.03	-0.15
AMH (ng/dL)	-0.4	0.18
Leptin (μg/ml)	-0.26	0.28

*p values indicate statistically significant (p<0.05).

PCOS, Polycystic ovarian syndrome; BMI, Body mass index; AFC, Antral follicle count; FSH, Follicle-stimulating hormone; LH, Luteinizing hormone; E2, Estradiol; TT, Total testosterone; 17OHP, 17-hydroxy progesterone; DHEA, Dehydroepiandrosterone; TSH, thyroid-stimulating hormone; AMH, Anti-Mullerian hormone.

## 4 Discussion

In times of nutritional deficiency, adipose tissue serves as a vital source of energy in addition to offering protection against mechanical harm and heat. The amount and distribution of adipose tissue are influenced by a number of significant factors, including food consumption, physical activity, and psychological variables (23). Numerous studies have revealed that leptin, the first adipokine, is involved in reproductive functions since its discovery (24–27). These results piqued curiosity about studying other adipokines, like adiponectin.

This study’s primary goal was to look into any potential relationships between women of reproductive age’s ovarian reserves and their levels of leptin and adiponectin. Based on their ovarian reserves, the women in this study were divided into three groups. The ovarian reserves were assessed using AFC, AMH, and FSH. Given the sensitivity of female reproductive health to age-related changes, particularly after 35 years and the average age of participants in group C is notably higher than that of the other two groups (**Figure 2A**), there was significantly lower AFC and AMH values and higher FSH values than the other two groups. In contrary, Group B had significantly higher AFC and AMH values than the other two groups, as would be expected.

Likewise, it was anticipated that Group B would have higher LH and androgen values because the pathophysiology of this syndrome is associated with these hormones’ elevated levels. Generally speaking, great care was taken to select women and assign them to the various study groups in compliance with globally recognized scientific standards. The primary constraint on this study is the comparatively small group sizes.

In our study leptin levels were increased in women of Group B compared to the other two groups without a statistical significance. However, leptin is affected by many different factors such as the distribution of body fat, fasting and the time of collecting the blood samples. In addition, we investigated the association of leptin with all parameters with each group. In group A and group B no significant statistical correlation of leptin with the other measured parameters was found. But in Group C a negative correlation was found between leptin and E2 and a positive correlation between leptin, AMH and adiponectin. The literature review did not provide any explanation for these findings, so they remain open to further investigation in the future.

Aiming to more thoroughly investigation we further sub-divided group B into two subgroups, based on BMI. In subgroup B1, a positive correlation of leptin with FSH and LH was found. LH is elevated in women with PCOS (28). Even today it is unclear what the role of leptin is in women with PCOS, although most researchers have found elevated leptin levels as well as leptin resistance in PCOS (both obese and lean subjects) (9, 29, 30). In subgroup B2, no statistically significant correlation was found between leptin and the remaining parameters.

Regarding adiponectin, the comparison of its values between the three groups (A, B and C) showed that group B had lower values compared to the other groups, but the difference was not statistically significant. Circulating adiponectin levels decrease with obesity and increase with weight loss (16). In our study group B had a statistically significant higher BMI than the other two groups.

In addition, we investigated the correlation of adiponectin with all parameters in each group. No statistically significant was found in group A. However, in group B, adiponectin was negatively correlated with LH and 17-OHP. As we know LH and 17-OHP are increased in PCOS (31). Also, most publications reported that adiponectin levels are decreased in women with PCOS (32–36). Therefore, we expected a correlation like this. In the subgroup B1 the same statistical result was found as we reported a negative correlation of adiponectin with LH and 17-OHP. No statistically significant correlation was found between adiponectin and parameters in subgroup B2. Finally, in the correlation analysis between adiponectin and Group C parameters, adiponectin was positively correlated with DHEA and leptin.

The main limitation of this study is the low sample number. Although there was no significant difference in leptin and adiponectin levels within the groups, due to the low sample number, it is difficult to generally conclude that leptin and adiponectin levels do not correlate with ovarian reserve. Furthermore, several factors determine plasma leptin and adiponectin levels which need to be dissected out in carefully controlled study design. As far as we know, there is no corresponding finding in the international literature, so further assays are needed.

## 5 Conclusion

In our study, leptin levels were elevated in women with PCOS who had an increased BMI, but there was no statistically significant difference compared to the other two groups. Leptin levels are affected by many different factors. Another possible explanation that there was no statistically significant difference between leptin and the three groups is that the number of samples was limited. Moreover, women with PCOS had lower adiponectin levels compared to the other two groups, but the difference was not statistically significant. The majority of studies found low adiponectin levels in women with PCOS regardless of BMI level although some studies have reported the exact opposite. Other factors that affect adiponectin levels are the body composition and the measured form.

In conclusion, we did not find compelling evidence linking circulating levels of leptin and adiponectin to ovarian reserves when we examined three groups of women of reproductive age (women with adequate ovarian reserves, women with high ovarian reserves, and women with diminished ovarian reserves). In our view, more research involving a greater number of participants is required to draw firm findings.

## Data availability statement

The original contributions presented in the study are included in the article/supplementary material. Further inquiries can be directed to the corresponding author.

## Ethics statement

The studies involving humans were approved by Research Ethics Committee of the Democritus University of Thrace (34792/223/13-2-2020). The studies were conducted in accordance with the local legislation and institutional requirements. The participants provided their written informed consent to participate in this study.

## Author contributions

KN: Writing – review & editing, Writing – original draft. NV: Writing – review & editing, Supervision. OP: Writing – review & editing. NN: Writing – review & editing, Supervision. KZ: Writing – review & editing. PT: Writing – review & editing. EK: Writing – review & editing. CD: Writing – review & editing. NG: Writing – review & editing. IP: Writing – review & editing, Data curation. BA: Writing – original draft, Writing – review & editing.
